# Supplementary material for: End Sequence Analysis Toolkit (ESAT) expands the extractable information from single-cell RNA-seq data
Source: Genome Res. 2016 Oct;26(10):1397–410. doi: 10.1101/gr.207902.116 (PMC5052061; doi:10.1101/gr.207902.116)
Supplement: Supplemental Material [file supp_gr.207902.116_Supp_Materials.tar.gz › ESAT-esat_v0.1/doc/html/classbroad_1_1core_1_1motif_1_1_position_weight_matrix_i_o.html]

ESAT: broad.core.motif.PositionWeightMatrixIO Class Reference


ESAT


- Main Page
- Related Pages
- Packages
- Classes
- Files

- Class List
- Class Index
- Class Hierarchy
- Class Members

All Classes Namespaces Files Functions Variables Enumerator Pages

- broad
- core
- motif
- PositionWeightMatrixIO

Public Member Functions |
Static Public Member Functions |
Static Public Attributes |
List of all members

broad.core.motif.PositionWeightMatrixIO Class Reference

Collaboration diagram for broad.core.motif.PositionWeightMatrixIO:

[legend]

|  |  |
| --- | --- |
| Public Member Functions | |
|  | PositionWeightMatrixIO () |
|  | |
| void | load (InputStream is) throws IOException, ParseException |
|  | |
| void | write (OutputStream os, NumberFormat formatter, List< PositionWeightMatrix > pwms) throws IOException |
|  | |
| void | writeUNIPROBE (OutputStream os, NumberFormat formatter, List< PositionWeightMatrix > pwms) throws IOException |
|  | |
| void | write (OutputStream os, NumberFormat formatter) throws IOException |
|  | |
| List< PositionWeightMatrix > | getMatrices () |
|  | |
| void | addPseudoCounts () |
|  | |

|  |  |
| --- | --- |
| Static Public Member Functions | |
| static void | main (String[] args) throws Exception |
|  | |

|  |  |
| --- | --- |
| Static Public Attributes | |
| static final String | USAGE |
|  | |

## Constructor & Destructor Documentation

|  |  |  |  |  |
| --- | --- | --- | --- | --- |
| broad.core.motif.PositionWeightMatrixIO.PositionWeightMatrixIO | ( |  | ) |  |

Here is the caller graph for this function:

## Member Function Documentation

|  |  |  |  |  |
| --- | --- | --- | --- | --- |
| void broad.core.motif.PositionWeightMatrixIO.addPseudoCounts | ( |  | ) |  |

|  |  |  |  |  |
| --- | --- | --- | --- | --- |
| List<PositionWeightMatrix> broad.core.motif.PositionWeightMatrixIO.getMatrices | ( |  | ) |  |

|  |  |  |  |  |  |
| --- | --- | --- | --- | --- | --- |
| void broad.core.motif.PositionWeightMatrixIO.load | ( | InputStream | *is* | ) | throws IOException, ParseException |

|  |  |  |  |  |  |
| --- | --- | --- | --- | --- | --- |
| static void broad.core.motif.PositionWeightMatrixIO.main | ( | String[] | *args* | ) | throws Exception |

static

Here is the call graph for this function:

|  |  |  |  |
| --- | --- | --- | --- |
| void broad.core.motif.PositionWeightMatrixIO.write | ( | OutputStream | *os*, |
|  |  | NumberFormat | *formatter*, |
|  |  | List< PositionWeightMatrix > | *pwms* |
|  | ) |  | throws IOException |

Here is the caller graph for this function:

|  |  |  |  |
| --- | --- | --- | --- |
| void broad.core.motif.PositionWeightMatrixIO.write | ( | OutputStream | *os*, |
|  |  | NumberFormat | *formatter* |
|  | ) |  | throws IOException |

Here is the call graph for this function:

|  |  |  |  |
| --- | --- | --- | --- |
| void broad.core.motif.PositionWeightMatrixIO.writeUNIPROBE | ( | OutputStream | *os*, |
|  |  | NumberFormat | *formatter*, |
|  |  | List< PositionWeightMatrix > | *pwms* |
|  | ) |  | throws IOException |

## Member Data Documentation

final String broad.core.motif.PositionWeightMatrixIO.USAGE

static

**Initial value:**

= "Usage: PositionWeightMatrixIO TASK=<task\_num> <task\_args> -pwm <PWM file in FASTA fromat>\n" +

"\tTasks:" +

"\n\t\t1. Report matches of a PWMs to a sequence -in <Sequence file or standard input in FASTA format> -pwm <File containing PWMs> -minScore <Minimum Score to report>" +

"\n\t\t\t-start <start matching at this point on the corresponding sequence include as many starts as sequenceIds> -end <Stop matching at this point on the corresponding sequence include as many ends as sequenceIds> " +

"\n\t\t\t[-seqId <Sequence Ids in the fasta file to search on specify as many as targets you want to search on, if non is specified all start/ends will be assume to relate to first sequence in file>] "+

"\n\t\tic Compute information content of all motifs on the file -pwm <File containing PWMs>" +

"\n"

---

The documentation for this class was generated from the following file:

- java/broad/core/motif/PositionWeightMatrixIO.java


---

Generated on Fri Aug 15 2014 15:10:54 for ESAT by  

 1.8.7
